# Impact of breast biopsy markers on magnetic resonance-guided focused ultrasound

**DOI:** 10.1080/02656736.2026.2632351

**Published:** 2026-02-23

**Authors:** Samuel I. Adams-Tew, Audrey Johnson, Joshua Crockett, Kate E. Adams, Dennis L. Parker, Nicole Winkler, Allison Payne

**Affiliations:** a Department of Radiology and Imaging Sciences, University of Utah, Salt Lake City, UT, USA; b Department of Biomedical Engineering, University of Utah, Salt Lake City, UT, USA

**Keywords:** Thermal ablation, MR thermometry, breast cancer, focused ultrasound, artifacts, metallic implants

## Abstract

**Background:**

The placement of a biopsy marker at the time of breast biopsy is standard of care to mark the biopsy site and facilitate surgical excision. However, their impact on incisionless treatments like magnetic resonance-guided focused ultrasound remains underexplored. These factors are important considerations for the design and execution of translational research in this field.

**Methods:**

Five commercially available markers were evaluated. Magnetic resonance imaging studies characterized artifact size across various treatment planning and monitoring sequences, as well as MR thermometry performance during focused ultrasound heating. Hydrophone scans collected acoustic field measurements in the presence of biopsy markers. Ablated volume in *ex vivo* tissue with and without markers present was estimated using measurements from thermal dose and gross tissue processing.

**Results:**

Substantial variability in artifact size and shape was observed across the markers. Thermometry showed both imaging and measurement artifacts, with off-target sites showing erroneous temperature change measurements of −30 to 100 °C in some cases. Acoustic field measurements revealed shape- and material-dependent distortions when markers were positioned near the ultrasound focus. Ablation experiments showed mixed effects on treatment volumes.

**Conclusion:**

The presence of biopsy markers introduces marker-dependent complexities into magnetic resonance-guided focused ultrasound treatment planning and monitoring. Reliable monitoring and energy delivery are achievable when the target is sufficiently far from the marker but become unreliable when the target is close to the marker. Accordingly, the potential impacts of biopsy markers on the safety and efficacy of MR-guided focused ultrasound treatments should be considered carefully.

## Introduction

Metallic markers are often placed during breast biopsies to mark the site for future reference and to confirm that the location of the biopsy performed matches the location of the original imaging finding [1,2]. If the site warrants surgical resection, the marker also facilitates localizing the area to be removed. When a surgical specimen is x-rayed, the presence of a marker allows immediate confirmation in the operating room that the target was resected. There is a spectrum of marker types approved to be placed in the breast. Commonly used markers are made of titanium or nitinol and are visible on mammography, ultrasound, and breast MRI, but cannot be detected externally without imaging. For these markers, if the area needs to be surgically removed, the patient must return for image-guided placement of a localizer. Markers that can both mark the site and serve as a localizer are being more commonly placed at the time of biopsy. These markers must be approved for indefinite implantation, and due to the increased cost relative to non-localizer markers, are only placed for lesions that have a high level of suspicion for breast cancer. Wireless localizers have become a popular alternative to the standard wire-guided localization for these cases [3,4]. These devices typically consist of a small metallic marker, sometimes embedded in a nonmetallic carrier material. They are often designed to create identifiable artifacts on ultrasound imaging or another localization modality which can guide surgical resection [5].

Although useful to mark an area biopsied to facilitate future surveillance or excision, these markers may have unwanted effects on magnetic resonance-guided focused ultrasound (MRgFUS) [2,6]. MRgFUS is in varying stages of technical development and clinical evaluation for breast cancer; the indication is generally not reimbursed by insurance, but it has received its first regulatory approvals in Europe and Asia [7]. Within the United States, clinical trials for the use of focused ultrasound in breast cancer treatment are ongoing, both for standalone and combination therapies [7]. Because placing markers during breast biopsy is part of the current standard of care, their presence must be carefully considered when evaluating FUS-based breast cancer therapies.

Biopsy marker literature primarily focuses on device safety, post-biopsy migration, and ultrasound and MR imaging impact [5,8–10]. The market offers a wide variety of markers: one retrospective review of mammograms conducted over a six-year period identified 38 different marker shapes from six manufacturers at a single tertiary cancer center [10]. Marker selection is thus largely determined by physician preferences in the various tradeoffs between these factors [5,10]. In MR imaging, metallic markers generally create signal voids at least twice the diameter of the marker itself and cause minor but clinically appreciable field inhomogeneity [9,11]. The application of fat saturation pulses also introduces additional clinically appreciable artifacts for some, but not all, clips [11]. The artifact from metallic markers also depends on orientation with respect to the main magnetic field, with the artifact being most prominent when the long axis of the marker is orthogonal to B_0_ [12].

There has been limited work evaluating the effect of biopsy markers and metallic fiducials on focused ultrasound therapies. One prior study has evaluated breast MRgFUS in the presence of breast biopsy markers [13]. That study evaluated the impact of markers on 2D MR temperature imaging during heating with focused ultrasound, concluding that the markers, despite having artifacts much larger than their actual size, had minimal impact on the volume of tissue above the thermal dose threshold [13]. Related work has evaluated focused ultrasound for prostate ablation in the presence of metallic fiducials for external beam radiation therapy (EBRT) [6,12,14]. Simulations of these treatments showed that the presence of a marker in the pre-focal region causes reflections, decreases focal intensity and volume, and results in a displacement of the focal spot, with the most substantial distortion occurring when the marker is within 5 mm of the intended focus [6]. A retrospective analysis of transurethral prostate cancer MRgFUS treatments in patients with EBRT fiducials in place observed a distinct lack of thermal dose accumulation behind markers [12]. MR imaging remained adequate for monitoring, with a strong agreement between thermal dose maps and contrast-enhanced post-treatment scans [12]. Studies in polyacrylamide hydrogel and bovine serum albumin phantoms observed that metallic markers substantially impacted the position and size of FUS ablation zones within 7 mm anteriorly, 18 mm posteriorly, and 3 mm laterally of the marker [14]. Targeting in front of the marker resulted in larger ablation zones, while targeting beyond the marker resulted in smaller ones [14]. MR artifacts and ultrasound beam distortion are known to occur in the presence of biopsy markers, but to our knowledge, no study to date has compared MR temperature imaging (MRTI) results to some non-MR ground truth.

It is expected that various markers will have different impacts on the MR imaging and FUS acoustic fields, depending on the marker’s materials, shape, and position relative to the region of interest. This study investigates how breast biopsy markers may influence thermally ablative MRgFUS breast cancer treatments by evaluating five commercial breast biopsy markers, including one radar-reflecting localizer, deployed in phantoms and *ex vivo* tissue. Experiments were conducted to characterize the impact of these markers on MR imaging and FUS acoustic fields, as well as the potential impact on real-time treatment monitoring and evaluation. MR experiments evaluated artifacts on several common sequences with markers embedded in tissue-mimicking phantoms and 3D temperature maps obtained during single-point sonications. Ultrasound experiments characterized the acoustic field using hydrophone measurements after transmission past a biopsy marker and in equivalent control conditions without a marker. Ablation studies were also performed in *ex vivo* porcine muscle with and without biopsy markers in place; following ablation, the tissue was grossly sliced for comparison between test conditions and with MRTI measurements during the ablation. This work provides greater insight into the potential impacts of biopsy markers on MRgFUS treatments. This understanding guides the design of clinical trials, informs the planning of treatments for patients with biopsy markers, and supports the evaluation of safety and efficacy in this context.

## Methods

**Figure 1.**
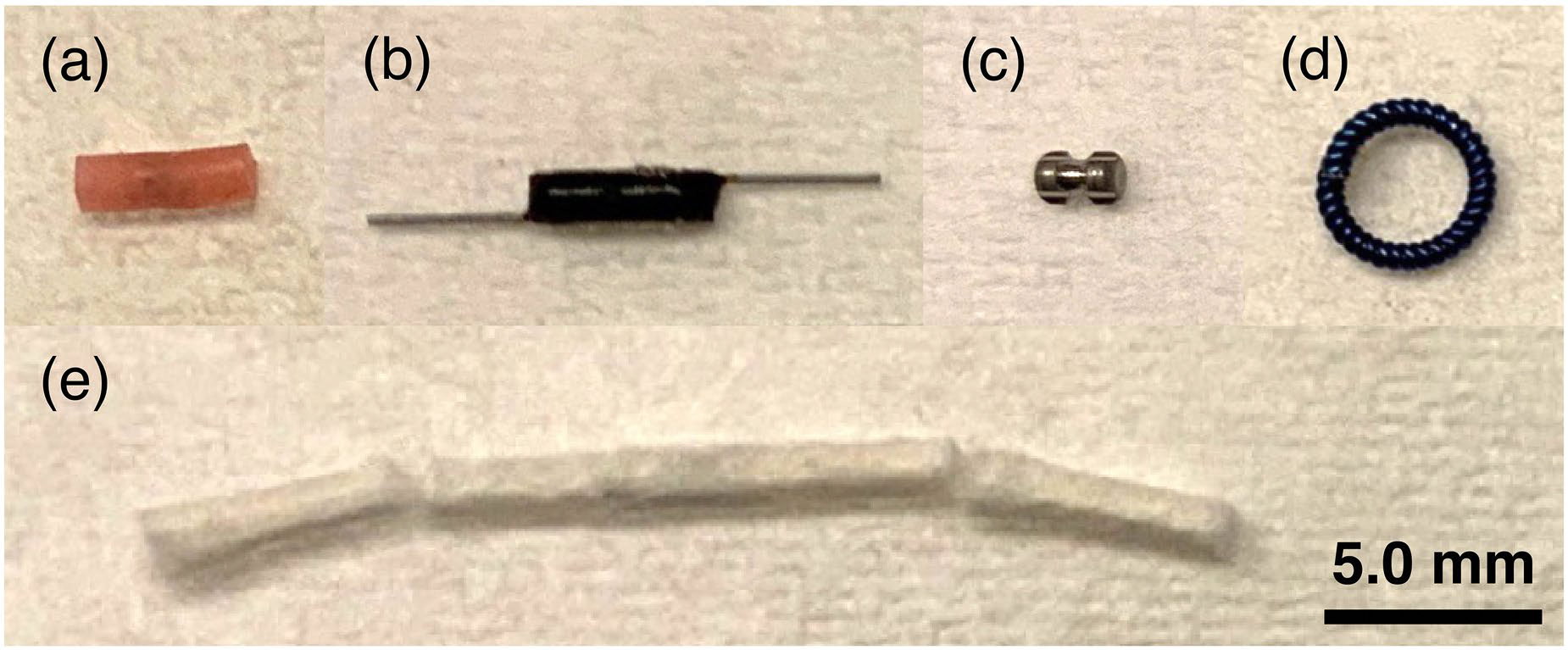
Images of biopsy markers used in this study. (a) HydroMark titanium marker embedded in hydrogel creates anechoic ultrasound artifact 24+ hours after implant; (b) Scout radar reflector with nitinol antennas; (c) TriMark titanium marker; (d) UltraCor Twirl self-embedding nitinol ring; (e) SenoMark UltraCor 108 marker with PVA padding resorbs into tissue over time.

Five breast biopsy markers representing a range of commercially available markers were used in this study (Figure 1): (a) an open coil titanium marker embedded in a hydrogel carrier (Mammotome HydroMark, 4010–02-15-T3; Devicor Medical Products, Cincinnati, Ohio, USA); (b) a radar reflecting localizer with nitinol antennas (Scout, SSR10–01 version J; Merit Medical Systems, South Jordan, Utah, USA); (c) an hourglass-shaped titanium marker (TriMark, TRIMARK-EVIVA-2S-13; Hologic, Marlborough, Massachusetts, USA); (d) a self-embedding nitinol ring (UltraCor Twirl, UCTW17; Bard Peripheral Vascular, Tempe, Arizona, USA); and (e) a BioDur 108 coil marker with PVA padding (SenoMark UltraCor, SMUC13C; Bard Peripheral Vascular). Of note, the radar reflector (b) is the only marker of the five that is also a localizer that can be detected by an external infrared probe.

A circular 1 MHz spherically focused phased-array transducer with 256 elements (13 cm focal length, 15.4 cm aperture, FWHM of free-field pressure profile 1.76 × 1.76 × 9.73 mm, fundamental frequency 1 MHz; Imasonic, Voray sur l’Ognon, France) was used for all experiments. Imaging and MR studies were performed in a 3 T Magnetom Vida or Prisma^FIT^ scanner (Siemens Healthineers, Erlangen, Germany).

### MR imaging and thermometry studies

Tissue-mimicking gelatin phantoms with and without markers embedded were used to evaluate marker impacts on imaging and MR thermometry. Cylindrical phantoms (6 cm diameter, 7 cm height) were prepared using a 50% milk-modified 250-bloom gelatin (Kirkland Evaporated Milk, Costco Wholesale Corporation, Issaquah, Washington, USA; Custom Collagen, Addison, Illinois, USA) described by Farrer et al. (speed of sound = 1549 m/s, acoustic attenuation = 0.54 dB/cm/MHz, thermal diffusivity = 0.143 mm^2^/s, specific heat capacity = 3635 J/kg/K) [15]. Biopsy markers were embedded by inserting them between layers during setting. Controls without a marker embedded were created under identical conditions at the same time as their counterparts.

After waiting ten days to ensure that marker carrier materials were fully hydrated, the phantoms were imaged in three orthogonal orientations. Follow-up images were acquired 50 days after phantom creation, following the phantoms’ use in MR temperature imaging experiments described below. Signal void artifacts were segmented using Seg3D [16] (version 2.5.1) by computing the mean of a no-signal region in or near the phantom, thresholding to 125% of that value, and selecting the contiguous region contained within the phantom. Artifact volume was computed from the number of voxels in each segmentation mask and the voxel size, whereas extent was computed from sample spacing and the maximum number of voxels along each imaging axis. Table 1 reports the imaging parameters and sequences used.

**Figure 2.**
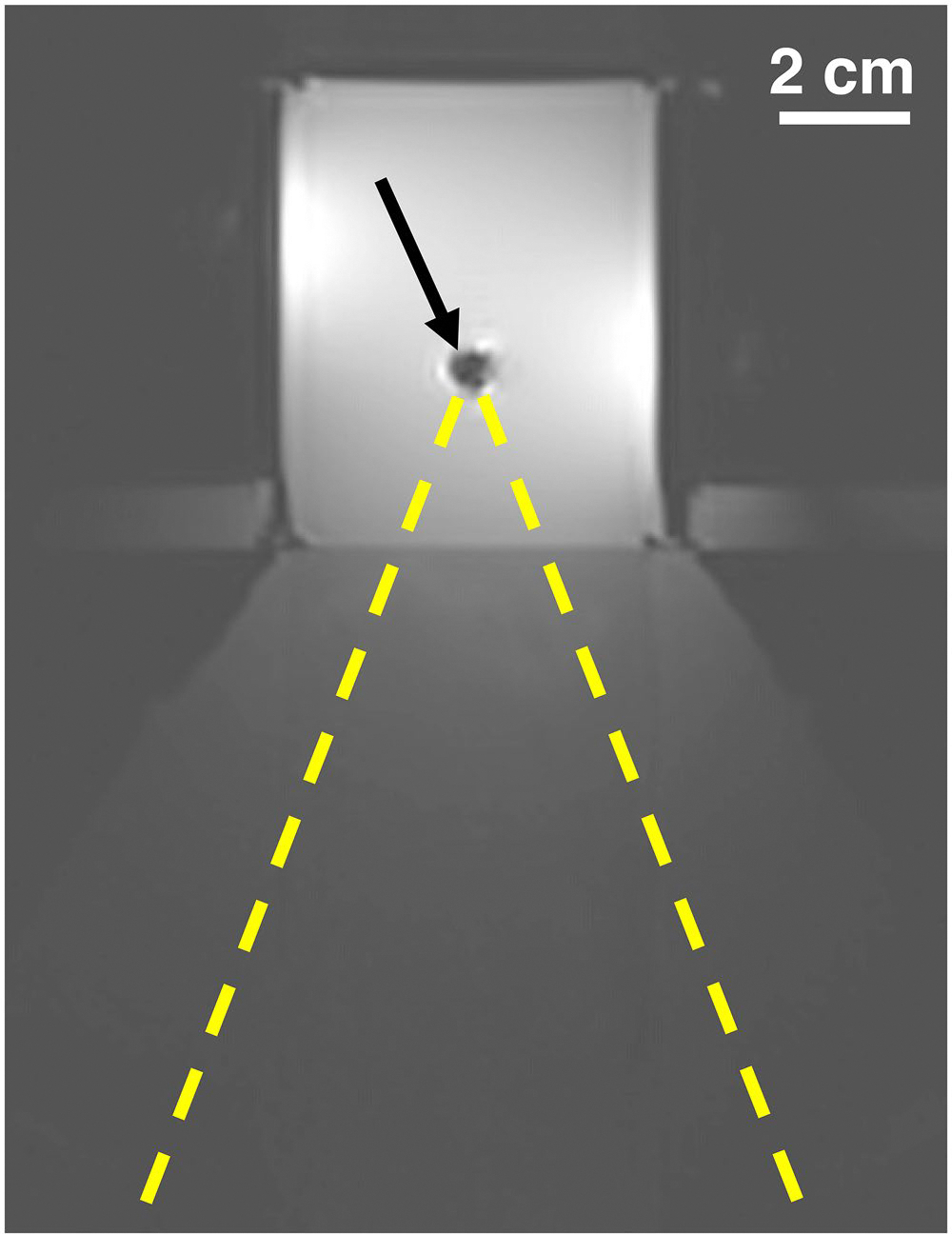
MRgFUS gelatin phantom experiment setup. TriMark biopsy marker is embedded in a phantom with acoustically absorbing gelatin. the marker’s signal void artifact is indicated by the arrow. The phantom rests over a water bath to acoustically couple the face of the phantom to a focused ultrasound transducer (out of figure at bottom, with approximate beam trajectory indicated by dashed lines). The same setup is also used for ablation experiments in pork loin.

In MR temperature imaging experiments, temperature change during FUS application was measured using a 3D MR thermometry sequence (2.16 s/dynamic; see Table 1 for other parameters) [17]. Two single-point sonications (each 24 acoustic W for 21 s) were performed for each biopsy marker, targeting the center of the signal void and a lateral edge of the artifact. The same electronic steering was applied to point sonications in corresponding controls. SNR masks were generated and applied to all images before evaluation. The analysis compared the peak temperature measurements and the similarity of temperature in equivalent voxels across both conditions for each marker. Because experiments occurred at room temperature, the total volume of all voxels within the phantom with a temperature rise greater than 10 °C was used as a proxy for treatment volume (note that this may be a noncontiguous volume). Figure 2 shows the MRgFUS experiment setup in gelatin phantoms.

### Acoustic field measurements

Thin-layer phantoms (7 cm radius, 20–35 ml gelatin) were created with 250 bloom gelatin, designed to hold the marker in place during sonication with minimal additional impact on the acoustic field. The biopsy marker was placed approximately at the center before allowing the gelatin to cool and set. For each condition, a control phantom was constructed from the same batch with the same quantity of gelatin.

**Figure 3.**
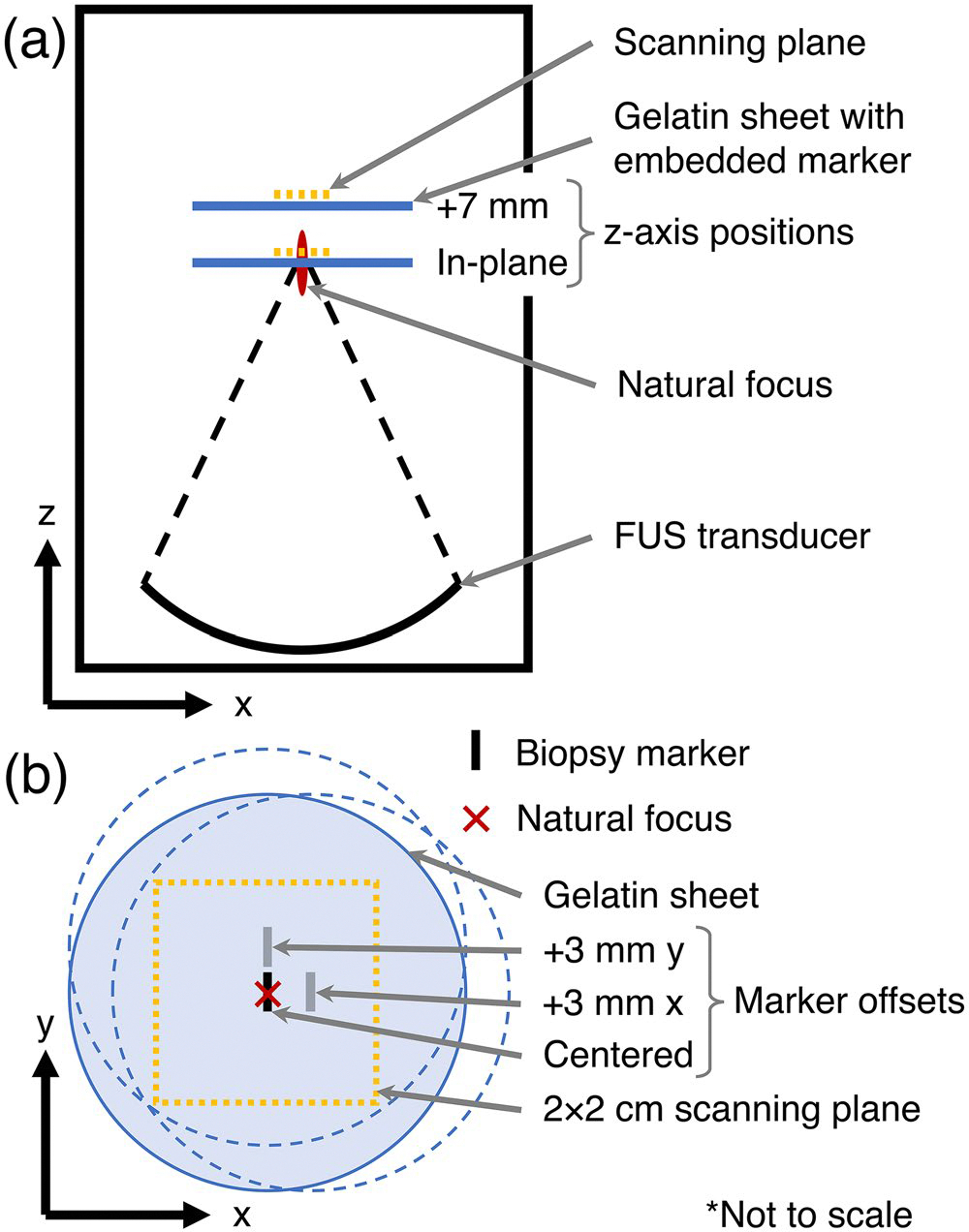
Schematic of experimental setup for acoustic field measurements using hydrophone. A biopsy marker embedded in a thin gelatin sheet was placed at one of six positions relative to the transducer’s natural focus. Acoustic field measurements were acquired in a 2 × 2 cm plane approximately 1 mm beyond the gelatin sheet using a hydrophone mounted on three axis stepper motors.

The acoustic field was measured using a vendor-calibrated hydrophone (HNR-0500, Onda Corporation, Sunnyvale, USA) and an in-house developed positioning system (NRT150 Linear Stage stepper motors; Thorlabs, Newton, New Jersey, USA). Figure 3 shows a schematic of the acoustic field measurement experiment setup, with all offsets determined relative to the natural focus. Biopsy markers were tested in two planes: in-plane with the natural focus and 7 mm far-field of the natural focus (Figure 3a). Within each plane, markers were placed in three positions: centered on the axis of propagation (at the natural focus for in-plane) and 3 mm lateral along each of the transverse axes (Figure 3b). For each marker position, hydrophone measurements were acquired in 2×2 cm planes (41×41 matrix, 0.5 mm spacing) centered on the axis of propagation, with the scanning plane approximately 1 mm behind the gelatin surface. Control scans with the gelatin-only counterparts were also acquired at each plane. Volumetric pressures over a 2×2×4 cm region around the natural focus were computed using the spatial planar projection method [18], validated as described below. Volumetric pressure patterns were normalized by the spatial peak pressure in the volume for the corresponding control, and peak pressure, focus volume (defined as the largest contiguous volume with amplitude greater than half the maximum), and focus displacement were characterized.

The accuracy of the spatial planar projection implementation for aberrated fields was evaluated using a set of 2×2 cm hydrophone scans (41×41 matrix with 0.5 mm sample spacing) at three offsets along the axis of propagation (no offset from natural focus, 6 mm near-field, 6 mm far-field). Angular spectrum calculations [18] were used to propagate each pattern into a 2×2×4 cm volume around the natural focus. The normalized pressure magnitude root-mean-squared difference (RMSD) throughout the volume was computed between the offset and no-offset conditions.

### Gross imaging comparison in pork loin

Ablation experiments were performed under MR guidance to target biopsy markers deployed in pork loin (Whole Foods Market, Inc., Austin, Texas, USA), with a single trial for each marker. Ablations consisted of 4–7 circular trajectories centered on or immediately adjacent to the signal void, using 98 to 115 acoustic W for 40 s. A limited number (*N* = 3) of sonications used 28 acoustic W for an initial confirmation of positioning and parameters. Volumetric MRTI measurements were acquired continuously during ablation and for 40 s following each sonication. A peripheral fiberoptic temperature probe provided a baseline temperature measurement for computing cumulative thermal dose. Control experiments were conducted by repeating the parameters of all sonications for a particular marker experiment in a separate cut of pork loin with no biopsy marker deployed. An SNR mask was applied to each MRTI acquisition, and the compiled MRTI measurements across all sonications for a particular condition were used to compute thermal dose as cumulative equivalent minutes at 43 °C (CEM43). The largest contiguous volume with a thermal dose of at least 240 CEM43 was used for comparison across conditions and with the gross imaging analysis.

**Figure 4.**
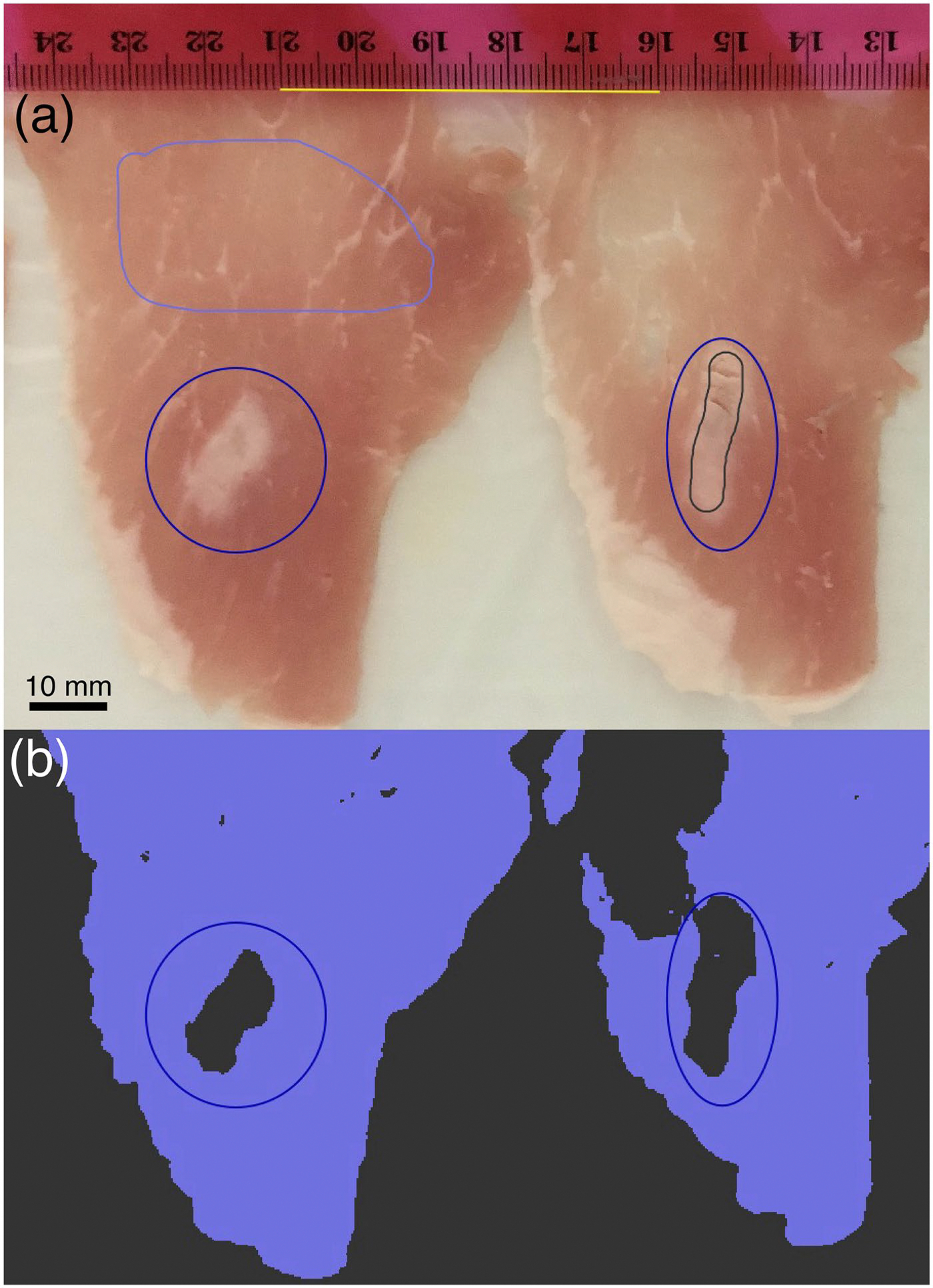
Sample of image annotation and measurement using QuPath. (a) Images of sliced ablated pork loin are annotated with samples of non-ablated (light blue) and ablated (gray) regions, and the pixel scale is set (yellow line). (b) A pixel classifier is trained to label each image as ablated and non-ablated. The area of the pixels annotated as ablated within the observer annotated regions of interest (blue ellipses) are recorded as the ablated area of each slice.

Following ablation, the pork loin was grossly sliced serially at approximately 1.5 mm thickness using a deli slicer. Each piece was laid flat and photographed, after which the thickness of any ablated region on each slice was measured using Vernier calipers (0.05 mm evident resolution). Images were annotated using QuPath [19] (version 0.4.3), and the ablated area in each image was computed (Figure 4) and combined with thickness measurements to estimate ablated volume. Combined standard uncertainty for the ablated volume in each trial was computed using the measured standard deviation of each component and assuming independence between components. Images were also reviewed to observe the shape of the ablation around each marker. Results from control and marker conditions were compared with each other and across MRTI and gross imaging.

## Results

**Figure 5.**
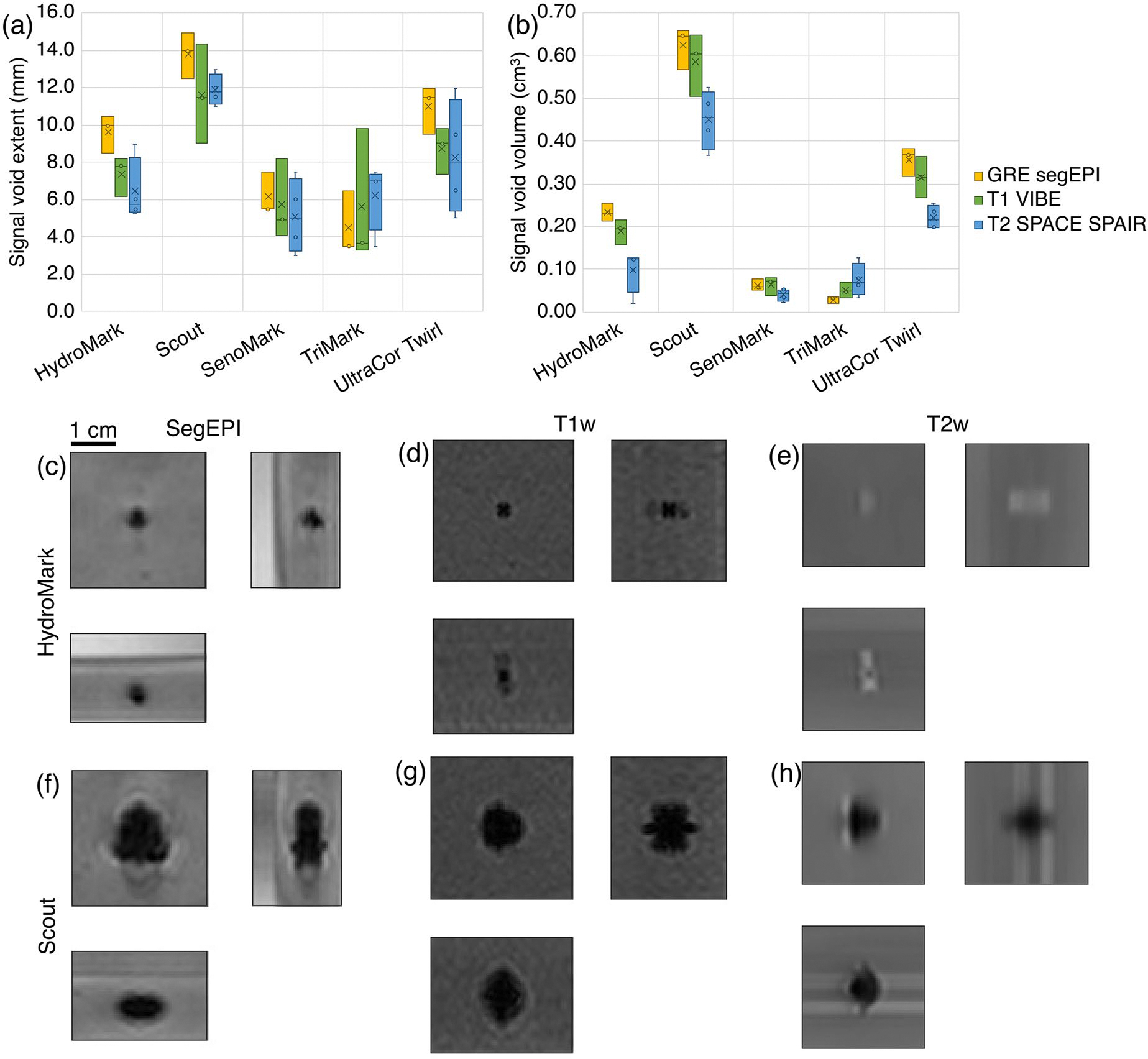
Box and whisker plots of plots of artifact size for the different markers. (a) Artifact extent along all three imaging axes (4 per axis, *N* = 12 total). (b) Overall signal void artifact volume (*N* = 4). Images of biopsy marker-induced signal void artifact for the HydroMark (c–e) and Scout (f-h) markers on segmented EPI (c,f), T1-weighted (d,g), and T2-weighted (e,h) MR imaging. Images for other markers available in supplementary material.

Imaging studies found large variations in artifact size and shape across markers. Across all studied biopsy markers, signal void extent varied from 2.7 to 12.0 mm in diameter, and the SenoMark and TriMark markers had the smallest artifact volumes overall (Figure 5a,b). Examples of artifacts from the HydroMark and Scout markers in a single imaging orientation for the GRE segmented EPI, T1-weighted, and T2-weighted scans are shown in Figure 5c–h. Orientation did not make a substantial difference in artifact shape or size; images of all markers in all orientations can be found in supplementary material Figures S1–S5. Because the orientation and positioning of the markers, as well as the precise clinical timing of the imaging, are not controllable, artifact variation with respect to orientation and days since embedding was not analyzed.

**Figure 6.**
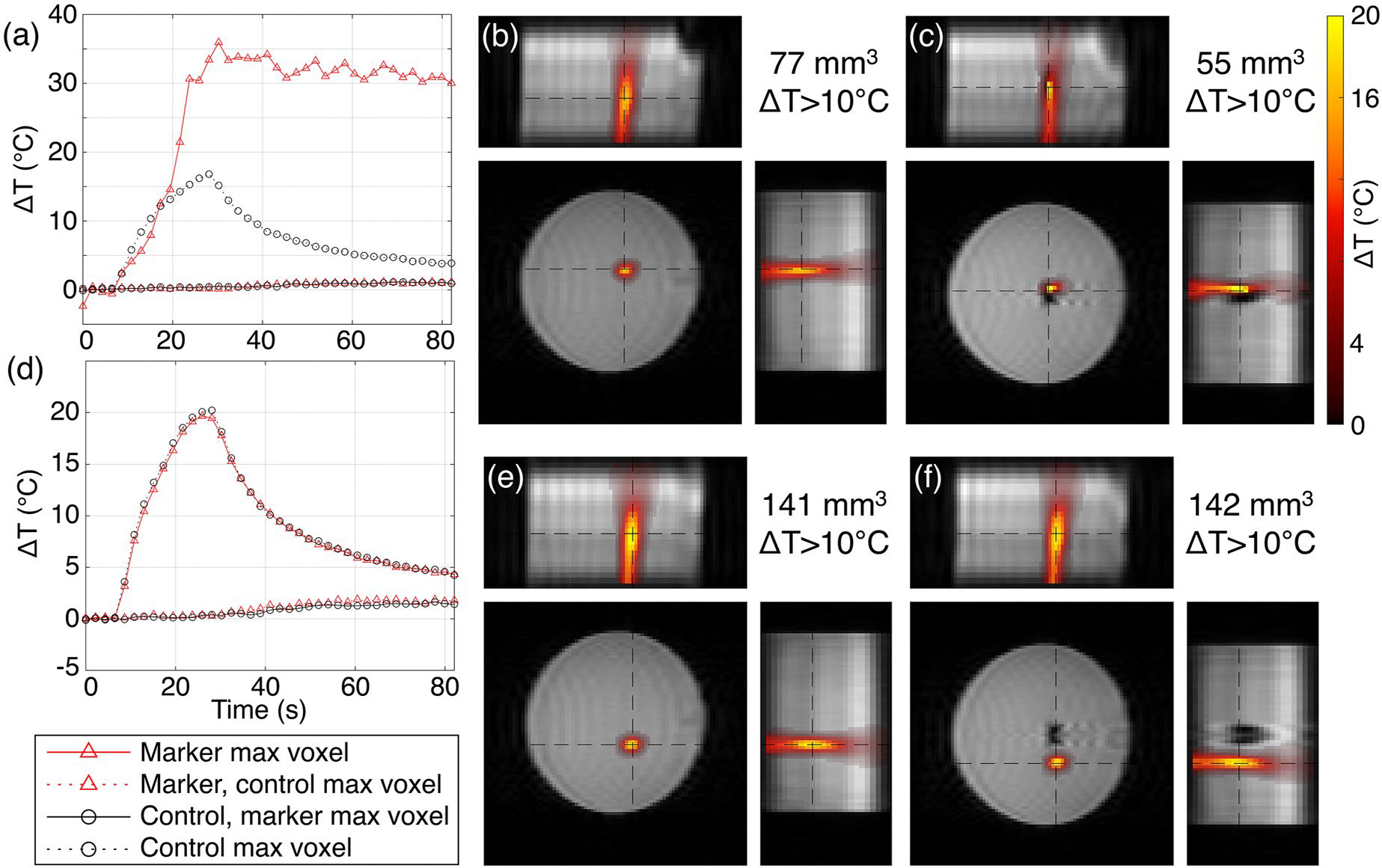
Measurements of focused ultrasound heating in an acoustically absorbing gelatin phantom with and without the HydroMark marker in place, targeting (a–c) the center of the signal void artifact and (d–f) a lateral edge of the artifact. Targeting was performed on images from a T1-weighted sequence with different artifact characteristics than the MRTI sequence. (a,d) Temperature change over time in a single voxel (the peak voxel with and without the marker in place). Temperature maps are shown for the time point with the highest heating during trials without (b,e) and with (c,f) the marker in place; the volume of voxels exceeding 10 °C temperature rise is reported in the upper right. Black dashed lines in the temperature maps indicate the slice positions for each displayed plane.

**Figure 7.**
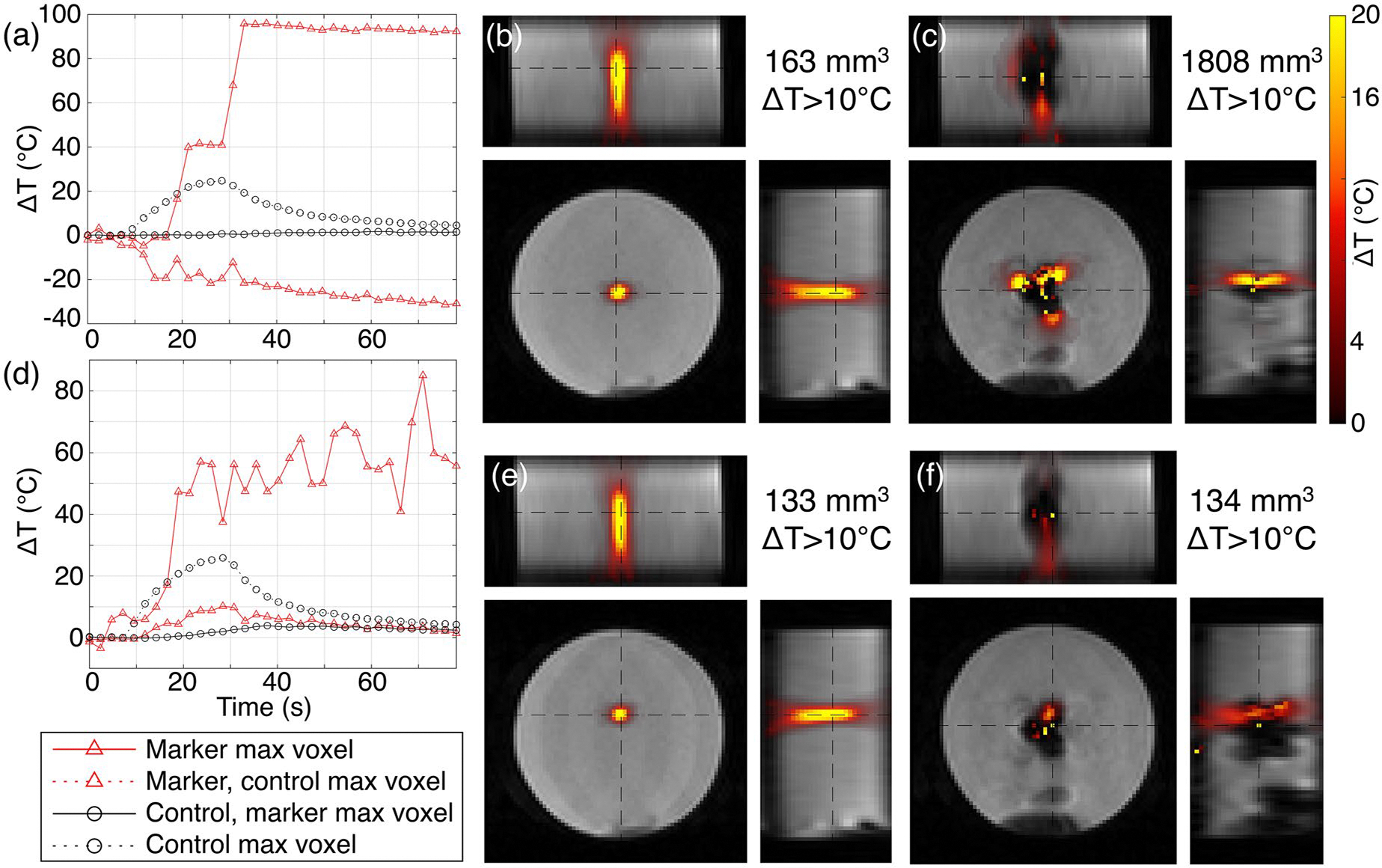
Measurements of focused ultrasound heating in an acoustically absorbing gelatin phantom with and without the Scout marker in place, targeting (a–c) the center of the signal void artifact and (d–f) a lateral edge of the artifact. Targeting was performed on images from a T1-weighted sequence with different artifact characteristics than the MRTI sequence. (a,d) Temperature change over time in a single voxel (the peak voxel with and without the marker in place). Temperature maps are shown for the time point with the highest heating during trials without (b,e) and with (c,f) the marker in place; the volume of voxels exceeding 10 °C temperature rise is reported in the upper right. Black dashed lines in the temperature maps indicate the slice positions for each displayed plane.

MR temperature imaging experiments evaluated the performance of temperature measurements during focused ultrasound heating near a biopsy marker. Figures 6 and 7 depict MRTI data from single-point sonications, together with volumes of at least 10 °C temperature rise, in tissue-mimicking gelatin phantoms for the control condition and with either the HydroMark or Scout marker in place (TriMark and UltraCor Twirl are shown in supplementary material Figures S6 and S7). For the HydroMark, targeting the lateral edge of the artifact gave measurements that agreed well with the paired control (Figure 6d–f), whereas targeting inside the artifact gave a visually interpretable but quantitatively nonphysical result (Figure 6a–c). For the Scout, MR thermometry showed unusual spatial and temporal features when targeting both positions (Figure 7). Targeting the center of the signal void artifact (Figure 7a–c) found time-series measurements of temperature change diverging to span from −30 to nearly +100 °C, with the temperature increases spatially confined to lobes along the direction of the main magnetic field. In the Scout and UltraCor Twirl markers, targeting the center of the signal void artifact resulted in substantially larger volumes with temperature change measurements greater than 10 °C, whereas the HydroMark and TriMark markers showed slightly smaller volumes. Targeting lateral to the artifact generally resulted in comparable volumes across all markers.

**Figure 8.**
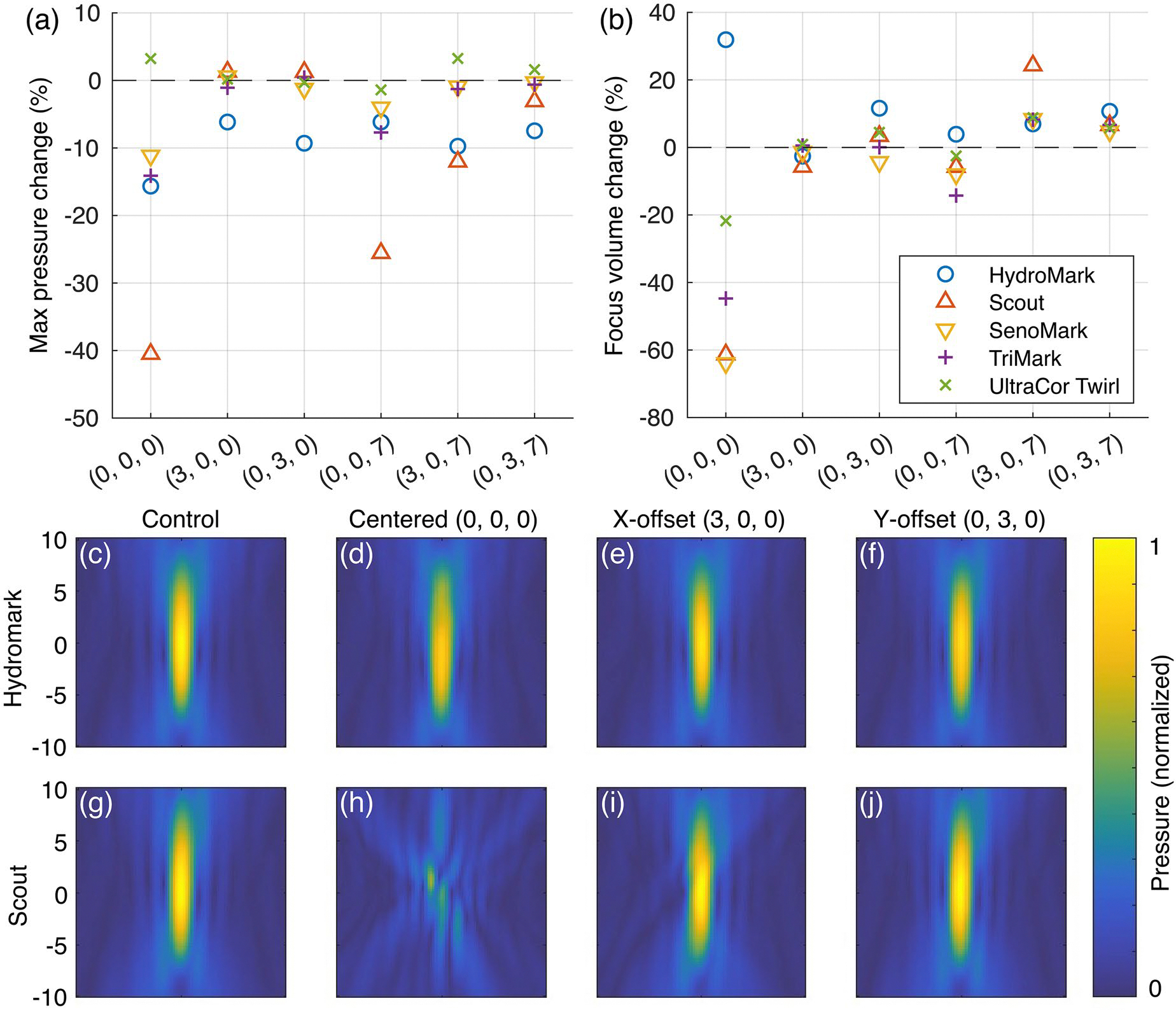
Hydrophone scan data sampled with and without a biopsy marker in the acoustic field. Biopsy markers are embedded in a thin (~2 mm) layer of gelatin. Plots show the change in maximum pressure (a) and focus volume (b) relative to the paired control for each marker at six positions relative to the natural acoustic focus. In the normalized pressure maps (c–j), each row depicts different marker offsets within the focal plane (see figure 3). the ‘control’ column (c,g) was acquired with no marker, only a thin layer of gelatin prepared from the same batch and at the same time as the respective samples. Centered (d,h), X-offset (e,i), and Y-offset (f,j) columns reference the position of the marker relative to the axis of propagation during data acquisition, as shown in figure 3. Pressure maps are normalized to the maximum pressure in the control volume for that row.

Acoustic field measurements with biopsy markers in place showed substantial aberration of the acoustic focus when the marker was positioned at the natural focus, with impacts being less pronounced with the marker in other positions. Figure 8a,b summarizes the change in maximum pressure and focus volume for all tested markers and positions. Figure 8c–j displays the x-z plane of the pressure field (normalized to the control) for the HydroMark and Scout markers positioned in-plane with the natural focus. Acoustic field maps for all markers and positions are available in supplementary material Figures S8 and S9, with plots of total focus displacement and displacement along the axis of propagation in Figure S8a,b.

Beam aberrations were most apparent when markers were placed at or behind the focus, with noticeable drops in peak pressure for all markers except the ring-shaped UltraCor Twirl (Figure 8a), and a decrease in focus volume for all markers except HydroMark (Figure 8b). Peak pressure varied substantially when a marker was positioned at the focus or at an offset along the axis of propagation (ranging across all markers from −41% to +3% of control for position [0, 0, 0] and −26% to −2% for [0, 0, 7]), with smaller effect when the clip was positioned with a 3 mm x- or y-offset from the focus (−12% to +3% range across all x- and y-offset conditions) (Figure 8a). For marker positions away from the natural focus (positions other than [0, 0, 0]), focus volume remained within ±20% of the control volume (excepting the Scout marker at +3 mm in x in the far-field plane), and varied substantially for all markers at the focus (range −64% to +32%). Focus displacement remained on the order of millimeters for all markers and positions, with displacement being largest for markers along the axis of propagation (Figure S8a,b). The largest total focus displacement observed for each marker was 0.5 mm for HydroMark, 1.3 mm for Scout, 0.6 mm for SenoMark, 1.0 mm for TriMark, and 0.2 mm for UltraCor Twirl (Figure S8b). In-plane insertion loss of measurements ranged from −0.30 to 4.0 dB. Validation of the spatial planar projection technique gave RMSD 4.5% and 4.7% when comparing acoustic field measurements derived from near- and far-field scans to the no-offset scans.

Ablations in pork loin were used to compare the measured ablation region determined using thermal dose from volumetric temperature imaging to volumes computed from annotated images of the gross tissue slices (Table 2). Thermal dose and gross evaluation of the ablation region agreed on the direction of relative change in the ablation region, though the direction was mixed across markers. For the HydroMark, SenoMark, and TriMark markers, the ablation volume was larger with the marker in place than without as evaluated by both methods. For the Scout and UltraCor Twirl markers, both techniques found that the ablation volume was smaller with the marker in place than without the marker.

## Discussion

Magnetic resonance-guided focused ultrasound (MRgFUS) is in active clinical trials for treating breast cancer in the United States and has received its first approvals in parts of Europe and Asia, necessitating operation within a clinical environment where biopsy markers are standard of care [7]. While identifying an ‘optimal’ marker was beyond the scope of this work, five clinically available markers were evaluated to investigate the range of effects on MRgFUS treatments [4,5,10]. The results illustrate the complexities that biopsy markers can introduce into MRgFUS treatment, including artifacts in planning and monitoring imaging and aberration of the FUS beam. The nature and magnitude of these effects depend on marker material, geometry, and orientation, which may not be known at the time of treatment. While reliable treatment appears achievable when the target is sufficiently far from the marker, the immediate vicinity of the marker presents distinct risks regarding energy delivery and monitoring accuracy.

### Imaging and treatment planning

Artifact size varied substantially across the evaluated markers and MR sequences tested. Markers showed a general trend for their relative artifact size (e.g., Scout generally has a larger artifact; SenoMark and TriMark, a smaller artifact) (Figure 5), but their performance varied such that no single sequence appears optimal for all markers. For example, while the signal void artifact volume for four markers was largest on the segmented EPI sequence, it was smallest for the TriMark on this sequence. This discrepancy poses a specific challenge for treatment planning and targeting, since an artifact may appear small on a planning sequence, but appear larger on the monitoring sequence. Furthermore, large artifacts from certain markers may preclude MR guidance altogether for the treatment of small tumors using current techniques.

### MR thermometry and treatment monitoring

Proton resonance frequency shift thermometry in the vicinity of nitinol markers (Scout and UltraCor Twirl) had unusual spatial and temporal features indicative of measurement artifact (Figures 7 and S7). The impacts likely stem from magnetic field distortions that are a function of the marker’s geometry, orientation, and magnetic susceptibility. When the precession frequency varies substantially across a single voxel, its signal is lost. Even outside the signal void, the spatially varying magnetic field results in a net change in the observed phase. This effect can be compounded by any temperature-dependent changes in the marker’s magnetic susceptibility when targeting a location that heats the marker (Figure 7a–c). As the temperature of the nitinol rises, its change in magnetic susceptibility introduces new magnetic fields that also contribute to the total observed external field. The net result is the appearance of heating and cooling in locations outside the signal void that may not be experiencing any substantial temperature change, together with the over- (or under-) estimation of the temperature changes that are occurring. Although not evaluated in this study, motion-coupled susceptibility changes can also result in large errors. This suggests that the development of temperature monitoring sequences for this environment will likely need to consider the impact of susceptibility not only on the image signal itself but also of temperature-dependent changes in susceptibility on the local magnetic fields.

### Thermal dose compared to gross pathology

Ablation in pork loin samples with and without biopsy markers present gave mixed results for the ultimate impact of markers on ablation (Table 2). In the TriMark experiment, the ablation size was approximately similar with and without the marker in place (0.84±0.26 vs. 0.79±0.20 cm^3^, respectively), though the thermal dose volume nearly doubled with the marker in place (1.80 vs. 0.95 cm^3^). For the remaining markers, treatment volumes were substantially different with a marker in place, as measured using both thermal dose and gross pathology. Interestingly, the two measurement methods were consistent with each other in determining the direction of change (if one method found a decrease in volume, the other did as well), but there was no apparent systematic bias in their disagreement (thermal dose-based ablation volume estimates can be larger or smaller than the volume estimated by gross measurement).

The finding that biopsy markers substantially impacted treatment volume contrasts with the previous work by Mougenot et al. who evaluated several MR-compatible gold, brass, and carbon-coated biopsy markers and found that they generally had minimal impact on the volume above the thermal dose threshold [13]. However, for the subset of markers where significant temperature differences were observed away from the marker, they also found a significant increase in the volume above the thermal dose threshold [13]. This suggests that the contrasting findings may be explained in part by the evaluation of a different set of markers and materials. It is also worth noting that the present study used 3D temperature imaging, which has intrinsically different volumetric characteristics, and 3 T scanners, where susceptibility artifacts generally have a greater spatial extent. These differences highlight the material-specific impacts of biopsy markers on MRgFUS, as well as the need to evaluate specific treatment pipelines and protocols with markers encountered at the study site.

### Acoustic fields and energy delivery

Each biopsy marker also had a unique impact on acoustic field measurements, depending on the position of the marker relative to the transducer’s natural focus and the makeup of that marker, which determines the acoustic impedance. Markers also have distinct shapes and sizes, as shown in Figure 1. Markers that approximate cylindrical solids and have a large acoustic impedance mismatch (Scout, SenoMark, TriMark) caused focus displacement and reduced peak pressure when positioned near the ultrasound focus. Qualitative observations during tissue slicing and measurement also corroborated previous findings of an acoustic shadowing effect in focused ultrasound treatments in the prostate when targeting regions located behind a metallic marker [12]. For the ring-shaped marker, however, positioning at the natural focus resulted in a greater focusing of the acoustic energy within the ring (Figure S9r). Importantly, relatively undistorted FUS beams could be achieved when the marker was positioned outside the full-width half-maximum boundary of the focus (rightmost two columns in Figures 8 and S8–S9), similar to the findings in [14]. Although the small titanium coil embedded in a hydrogel (Hydromark) resulted in small changes regardless of position (Figure 8a–f), the impacts of reflection and refraction might be greater in the actual use case, given that the impedance mismatch of hydrogel and tissue is greater than that of hydrogel and gelatin. Though outside the scope of this paper, full-wave acoustic simulations could provide additional insights into the impacts of marker material and orientation on focused ultrasound fields.

It is likely that the dramatic difference in acoustic properties between biopsy markers and the surrounding tissue results in enhanced heating around the marker. Such an effect would be analogous to the unwanted heating and burns that can accumulate during FUS treatments near bone [20,21]. The experimental endpoints evaluated in this study did not directly observe this effect, but that does not mean it is not occurring. The dramatic difference in properties between water and the biopsy marker forms the basis for the acoustic reflection and diffraction observed in the acoustic field measurements shown in Figure 8. It is possible that, because of the small size of both the ultrasound focus and the biopsy marker, any increased heating is highly localized to the area around the marker, not observable using MRI because of the artifact and not obvious from the binary classification of dissected tissue. For hyperthermia treatments, such increased and localized heating introduces a risk of unintentionally ablating tissue. Conversely, ablative therapies may be more efficient in ablating the tissue immediately surrounding the marker, such that findings of treatment efficacy necessitate careful interpretation to properly generalize to the marker-free environment. Both risks should be considered when planning a study and carrying out treatments in the immediate vicinity of a marker.

### Considerations for practitioners

The experiments in this work were conducted in phantoms and *ex vivo* tissues, which cannot fully reproduce physiological effects such as perfusion and edema or the full *in vivo* tissue-marker interactions. The results nonetheless highlight several physical and practical considerations for researchers conducting MRgFUS in the presence of biopsy markers:

1. Acoustic fields are dramatically disrupted when the marker falls along the axis of propagation of the ultrasound beam. The resulting reflections and distortions are a function of the marker materials, orientation, and geometry, making the field unpredictable in the current clinical environment. Over- and under-treatment can occur immediately around the marker due to reflections and shadowing, which may be of particular concern for applications that use predefined trajectories with little real-time monitoring.
2. Minimal disruption of the acoustic field occurs when targeting positions lateral to the marker. This appears to hold true for cases where the marker was positioned outside the full-width half-maximum of the ultrasound beam (Figures 8 and S8–S9, rightmost two columns). Greater clearance may be necessary in practice, however, due to measurement artifacts in MR thermometry.
3. Proton resonance frequency shift MR thermometry shows both imaging and measurement artifacts. When heating very close to a marker, temperature-dependent changes in the marker’s magnetic susceptibility can lead to observations of heating and cooling that are not truly present, even in regions outside the signal void artifact (Figure 7). Leaving a small margin (millimeters) between the ultrasound focus and the signal void appears to give reliable measurements (Figure 6d–f).
4. Clear documentation of marker shapes, materials, and placement would greatly benefit the MRgFUS research community in further understanding the challenges posed by biopsy markers and developing appropriate strategies for conducting treatments and clinical trials.

## Conclusion

The presence of breast biopsy markers introduces variable and complex disruptions to current MRgFUS workflows. Of the markers evaluated here, the least disruptive marker was the Hydromark (a small titanium coil embedded in a hydrogel), and the most disruptive was the Scout (a radar reflector with nitinol antennas), with the impact of other markers falling somewhere between these two, and all markers influencing imaging and energy delivery to some extent. Importantly, reliable monitoring and energy delivery appear achievable using current technologies when the region of interest is sufficiently far from the biopsy marker. Within the immediate vicinity of a marker, however, the impact of MR and FUS artifacts and distortions on a study’s specific safety and efficacy questions needs to be carefully considered to mitigate any unique risks and understand the generalizability of the study’s findings. While the presence of biopsy markers introduces challenges to these treatments and studies, this work supports an understanding of those challenges and the development of strategies to work safely and effectively within that environment.

## Supplementary Material

Supp 1

Supplemental data for this article can be accessed online at https://doi.org/10.1080/02656736.2026.2632351.

## Figures and Tables

**Table 1.** MRI sequences and parameters.

Sequence	TR (ms)	TE (ms)	Field of view (mm)	Voxel size (mm)	FA (°)

3D T1 VIBE	7.4	4.7	262×156×88	0.4 × 0.4 × 1	10
T2 SPACE SPAIR	1200	121	224×159×80	0.6 × 0.6 × 1	120
GRE seg EPI (imaging)	36	12	224×154×30	0.6 × 0.6 × 3	20
GRE seg EPI (MRT)	36	12	192×84×24	1×1×3	13

**Table 2.** Ablation size with and without a biopsy marker in place as assessed by MR thermometry and gross pathology measurements in an *ex vivo* pork loin experiment. MR thermal dose measurements are CEM 43 °C. Gross pathology measurements are derived mean ± combined standard uncertainty.

	Measured ablated volume (cm^3^)			
	
	MR thermal dose		Gross pathology	
		
Marker	Control	Marker	Control	Marker

UltraCor Twirl	1.60	2.54	1.39 ± 0.22	3.86 ± 1.25
HydroMark	1.05	0.46	0.78 ± 0.16	0.27 ± 0.10
TriMark	1.16	4.29	1.10 ± 0.25	3.29 ± 1.21
SenoMark	0.95	1.80	0.79 ± 0.20	0.84 ± 0.26
Scout	2.74	1.43	1.60 ± 0.47	0.57 ± 0.07

## Data Availability

The data that support the findings of this study are available from the corresponding author, SIAT, upon reasonable request.
