# Supplementary material for: Impact of breast biopsy markers on magnetic resonance-guided focused ultrasound
Source: Int J Hyperthermia. Author manuscript; Available in PMC 2026 Apr 13. (PMC13074824; doi:10.1080/02656736.2026.2632351)
Supplement: Supp 1 [file NIHMS2153872-supplement-Supp_1.docx]

**Impact of breast biopsy markers on magnetic resonance-guided focused ultrasound breast cancer treatments – supplementary figures**

**
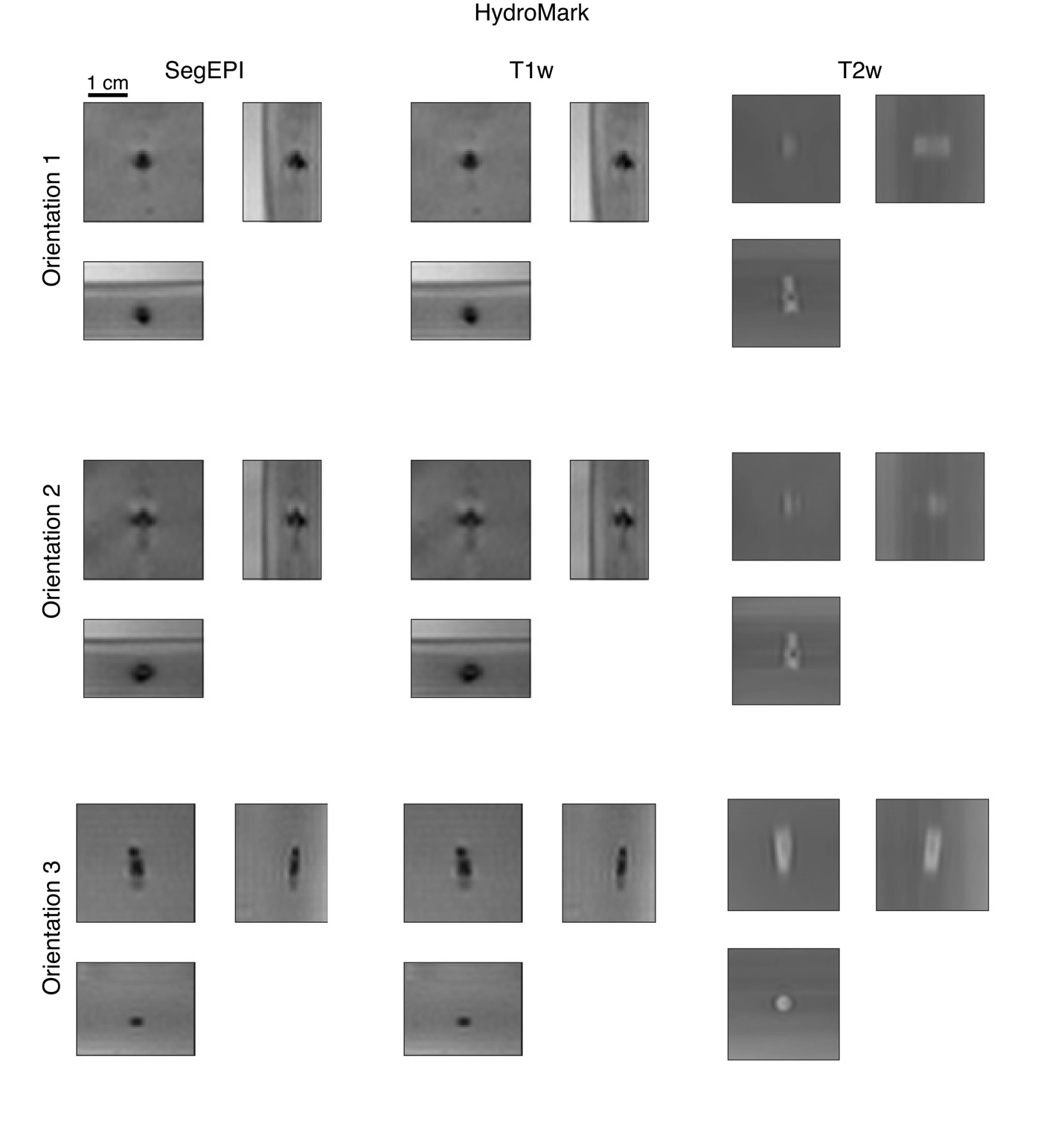
**

**Figure S1.** Images of biopsy marker-induced signal void artifact for the HydroMark marker on segmented EPI, T1-weighted, and T2-weighted MR imaging, with the marker in three different orientations relative to the main magnetic field.

**
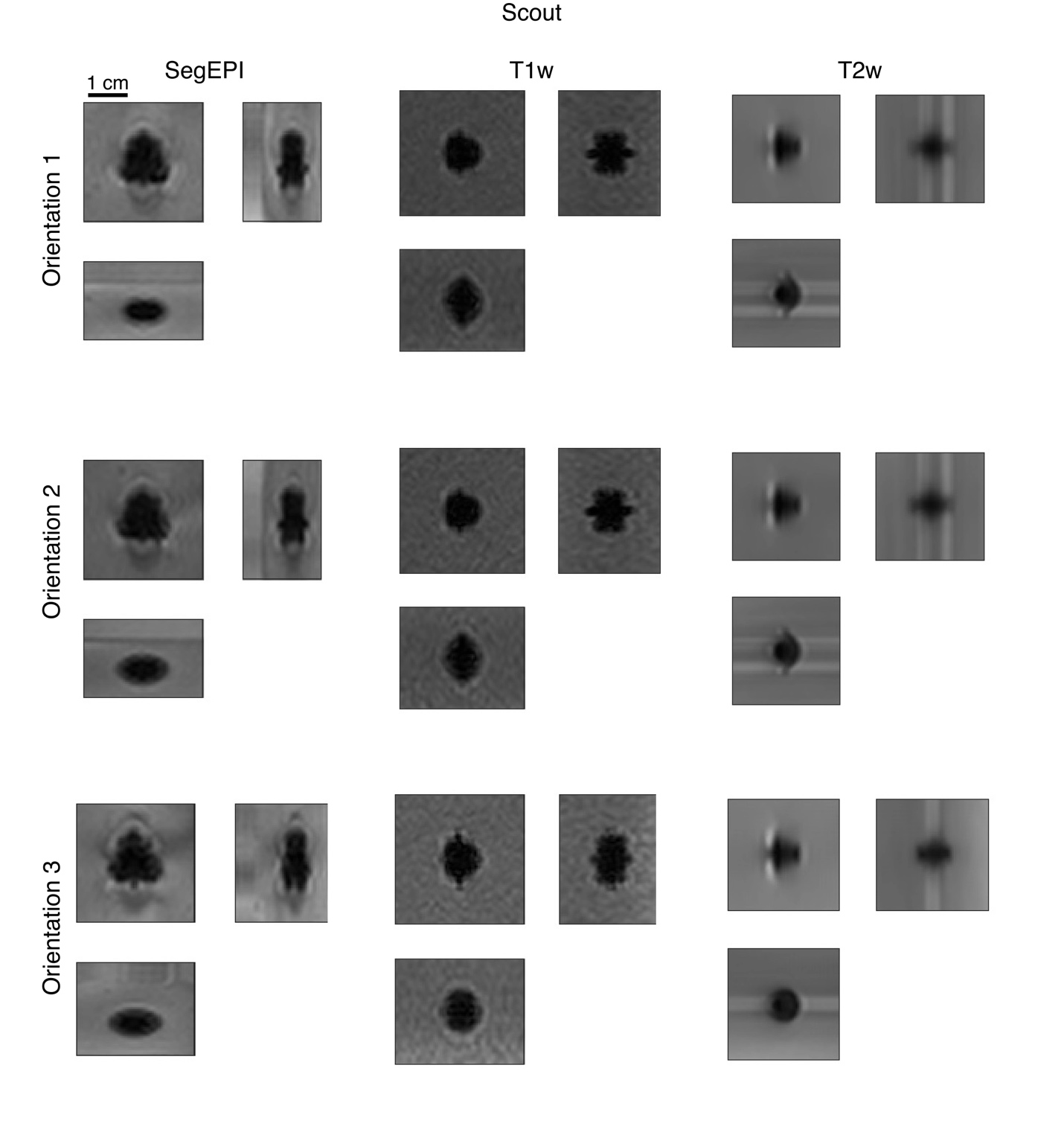
**

**Figure S2.** Images of biopsy marker-induced signal void artifact for the Scout marker on segmented EPI, T1-weighted, and T2-weighted MR imaging, with the marker in three different orientations relative to the main magnetic field.

**
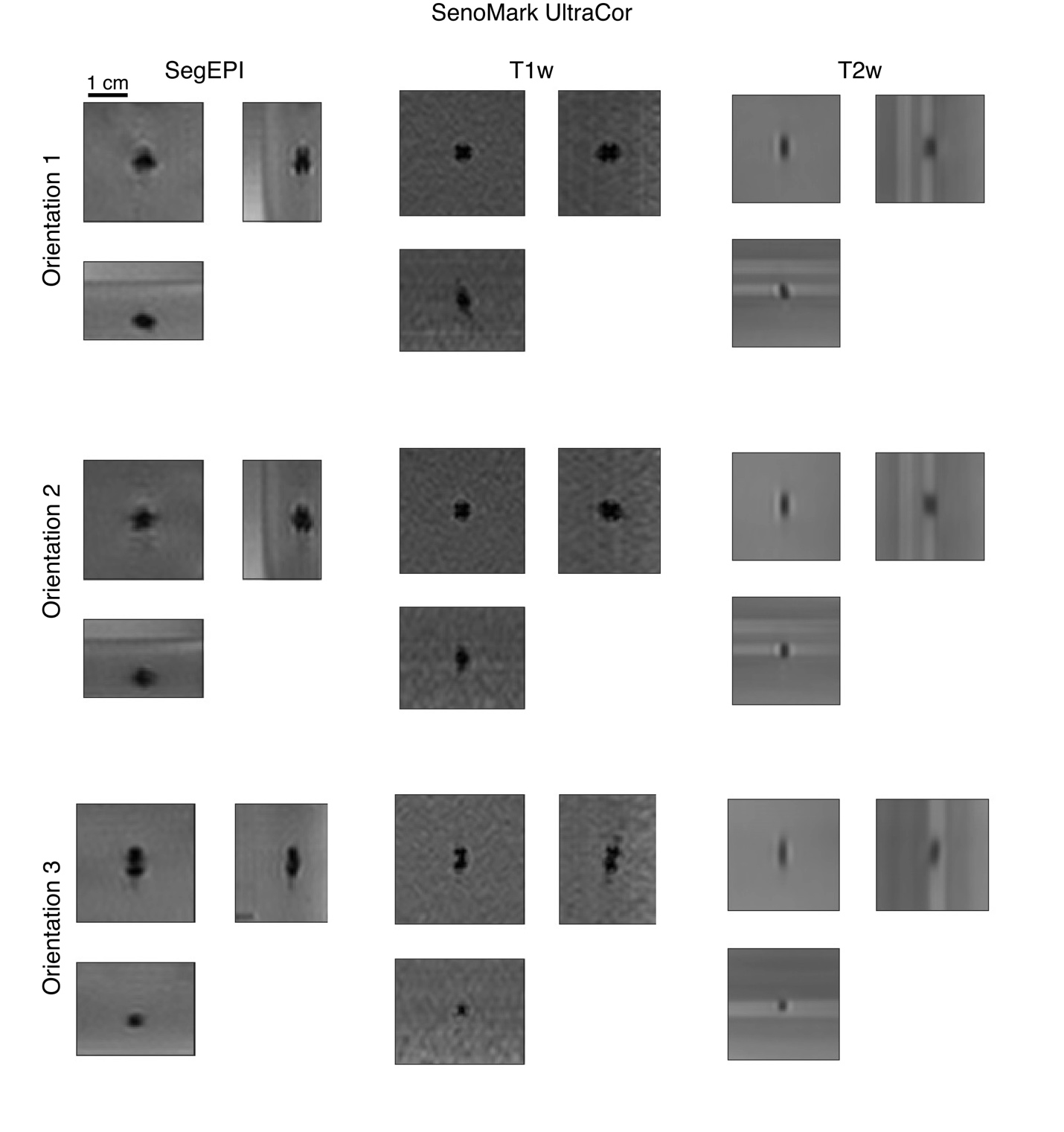
**

**Figure S3.** Images of biopsy marker-induced signal void artifact for the SenoMark UltraCor marker on segmented EPI, T1-weighted, and T2-weighted MR imaging, with the marker in three different orientations relative to the main magnetic field.

**
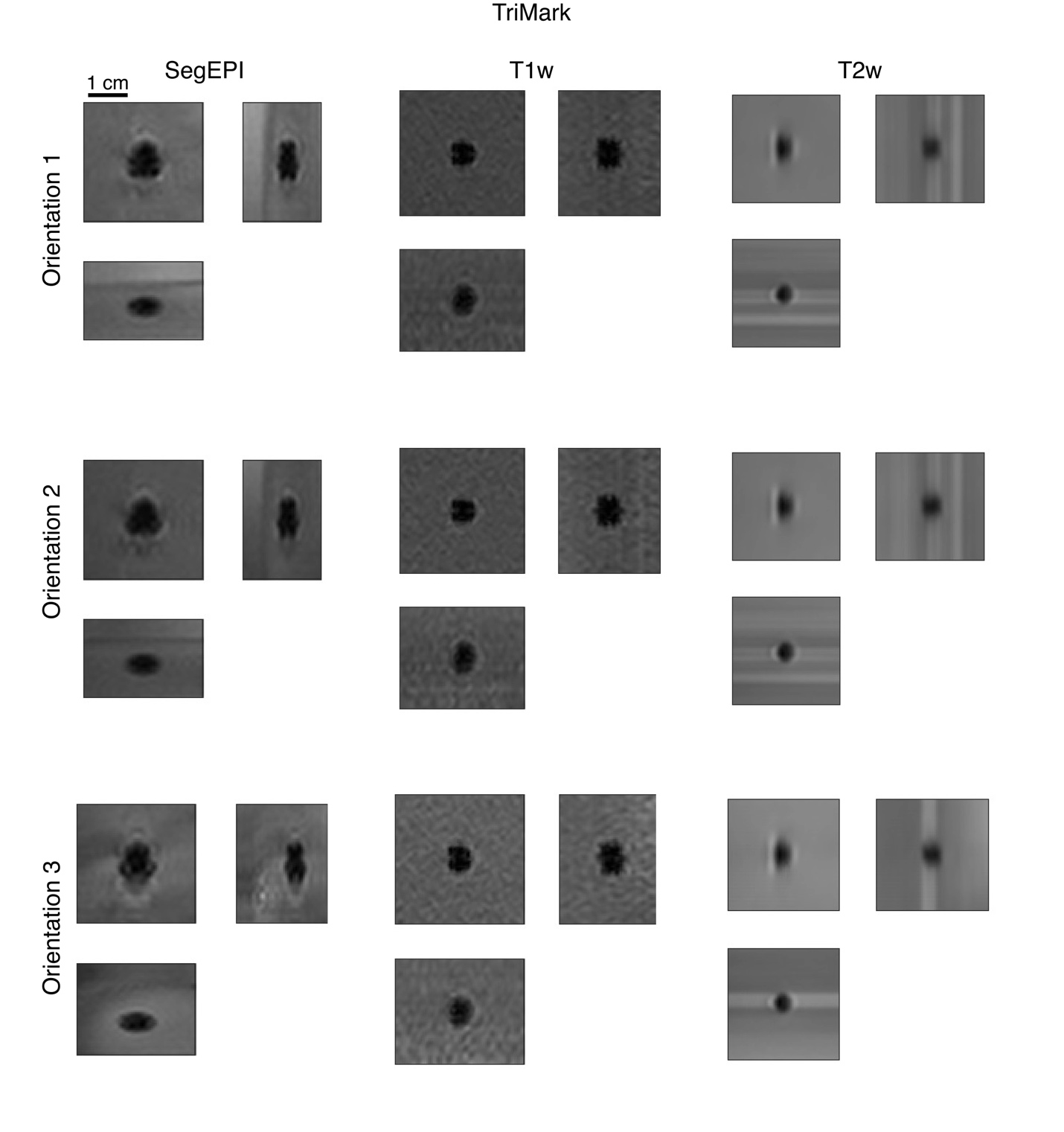
**

**Figure S4.** Images of biopsy marker-induced signal void artifact for the TriMark marker on segmented EPI, T1-weighted, and T2-weighted MR imaging, with the marker in three different orientations relative to the main magnetic field.

**
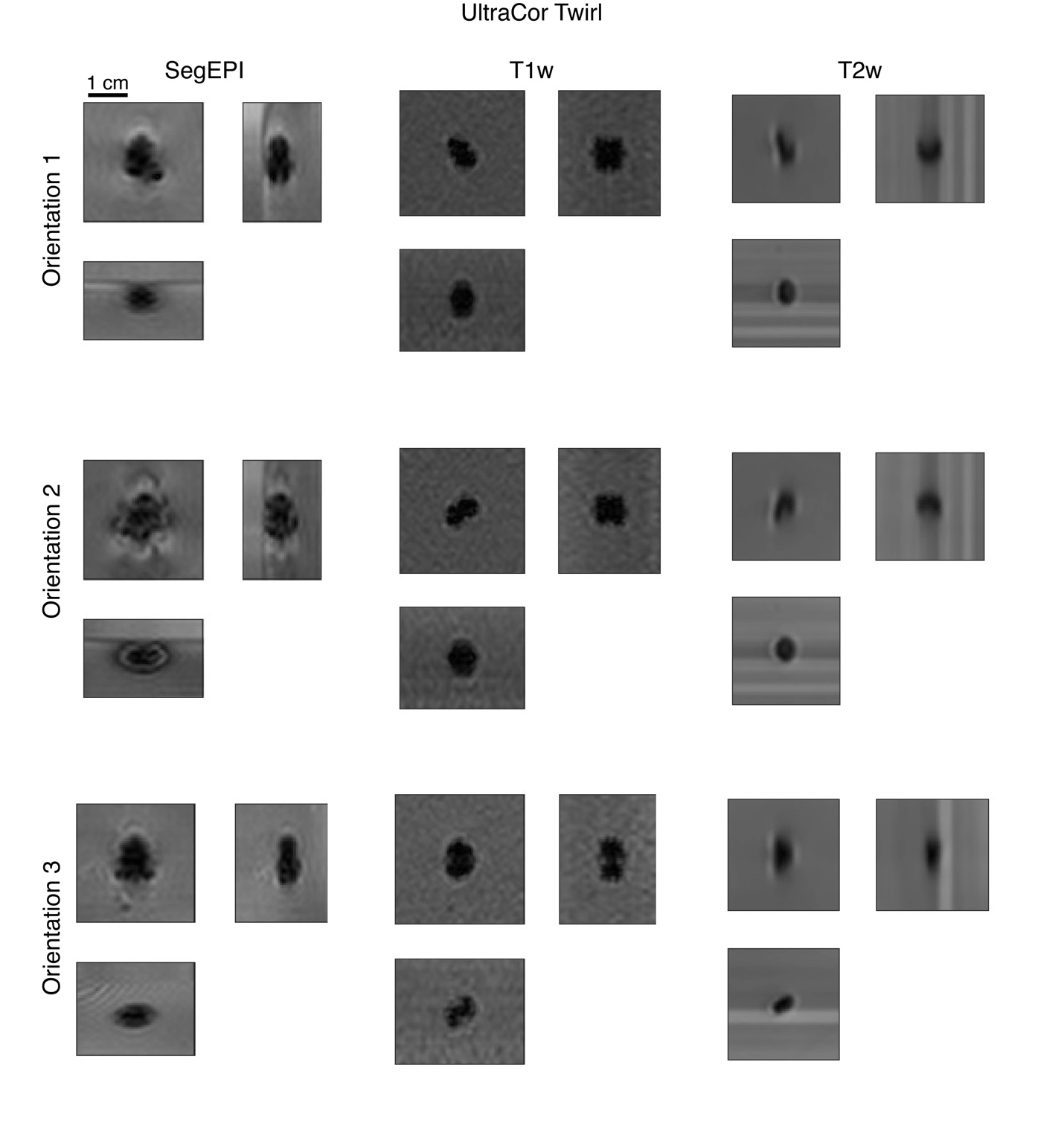
**

**Figure S5**. Images of biopsy marker-induced signal void artifact for the UltraCor Twirl marker on segmented EPI, T1-weighted, and T2-weighted MR imaging, with the marker in three different orientations relative to the main magnetic field.

**
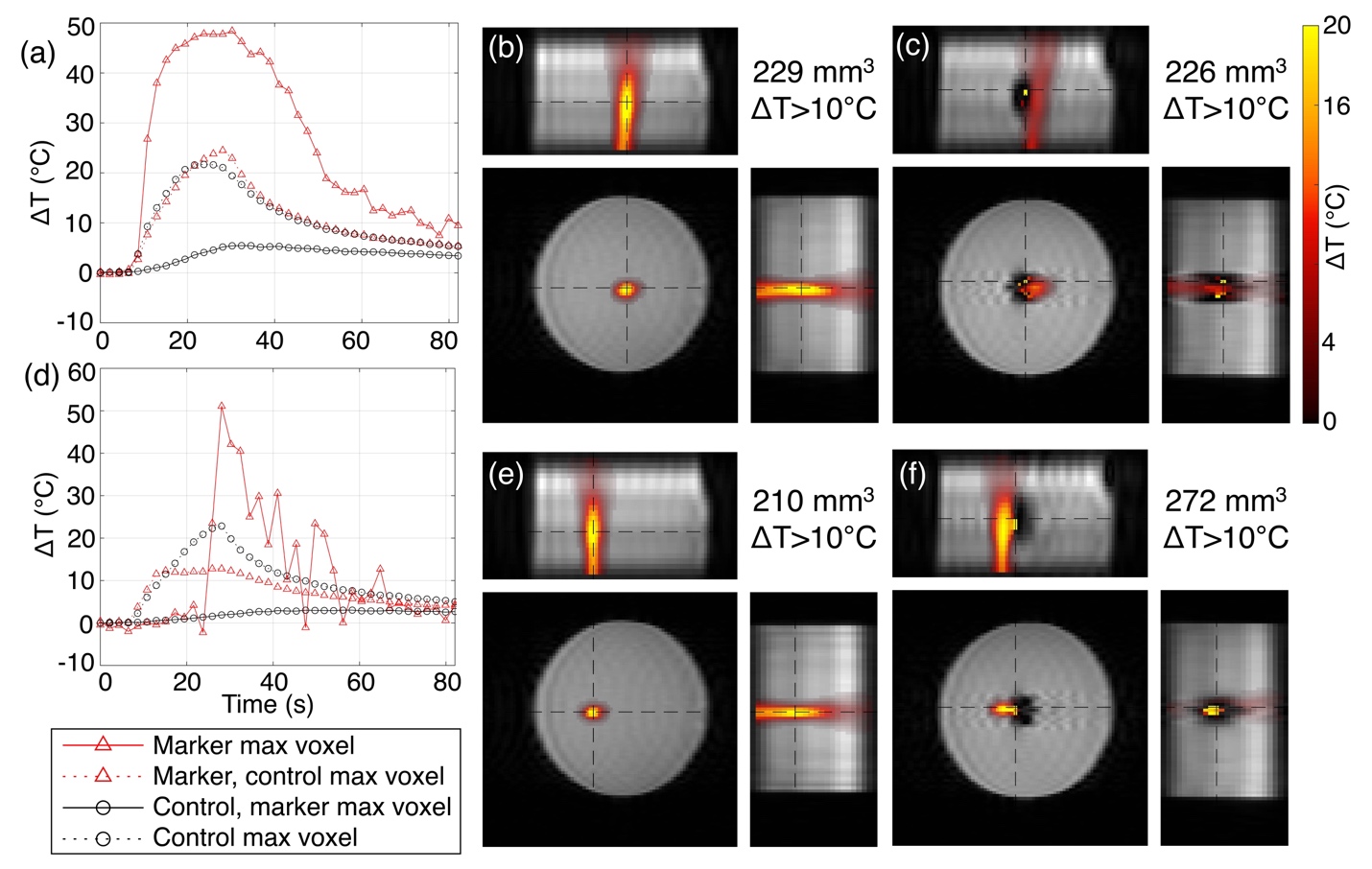
**

**Figure S6.** Measurements of focused ultrasound heating in an acoustically absorbing gelatin phantom with and without the TriMark marker in place, targeting (a-c) the center of the signal void artifact and (d-f) a lateral edge of the artifact. (a,d) Temperature change over time in a single voxel (the peak voxel with and without the marker in place). Temperature maps are shown for the time point with the highest heating during trials without (b,e) and with (c,f) the marker in place. Black dashed lines in the temperature maps indicate the slice positions for each displayed plane. (Compare Figs. 6-7)

**
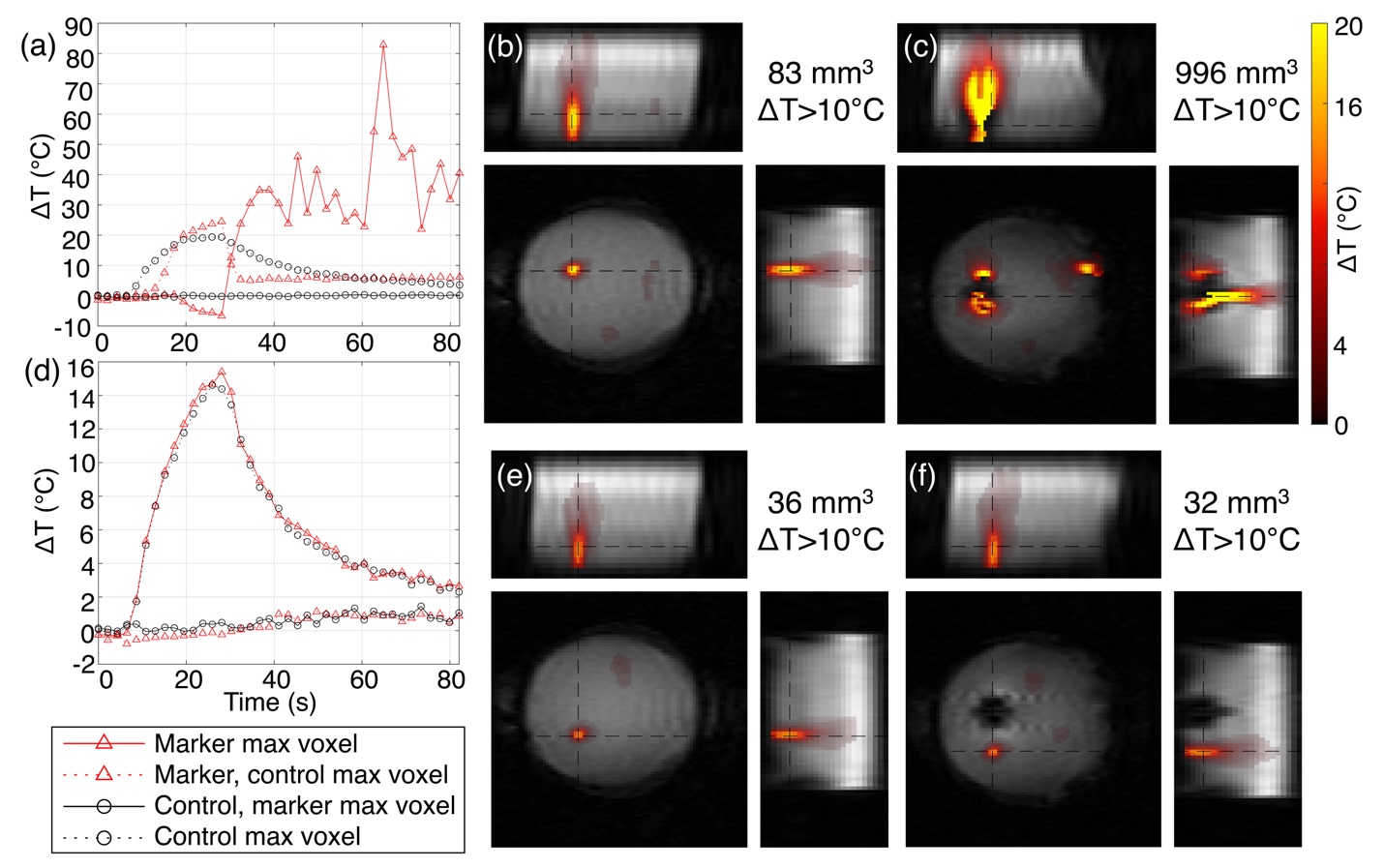
**

**Figure S7.** Measurements of focused ultrasound heating in an acoustically absorbing gelatin phantom with and without the UltraCor Twirl marker in place, targeting (a-c) the center of the signal void artifact and (d-f) a lateral edge of the artifact. (a,d) Temperature change over time in a single voxel (the peak voxel with and without the marker in place). Temperature maps are shown for the time point with the highest heating during trials without (b,e) and with (c,f) the marker in place. Black dashed lines in the temperature maps indicate the slice positions for each displayed plane. (Compare Figs. 6-7)

**
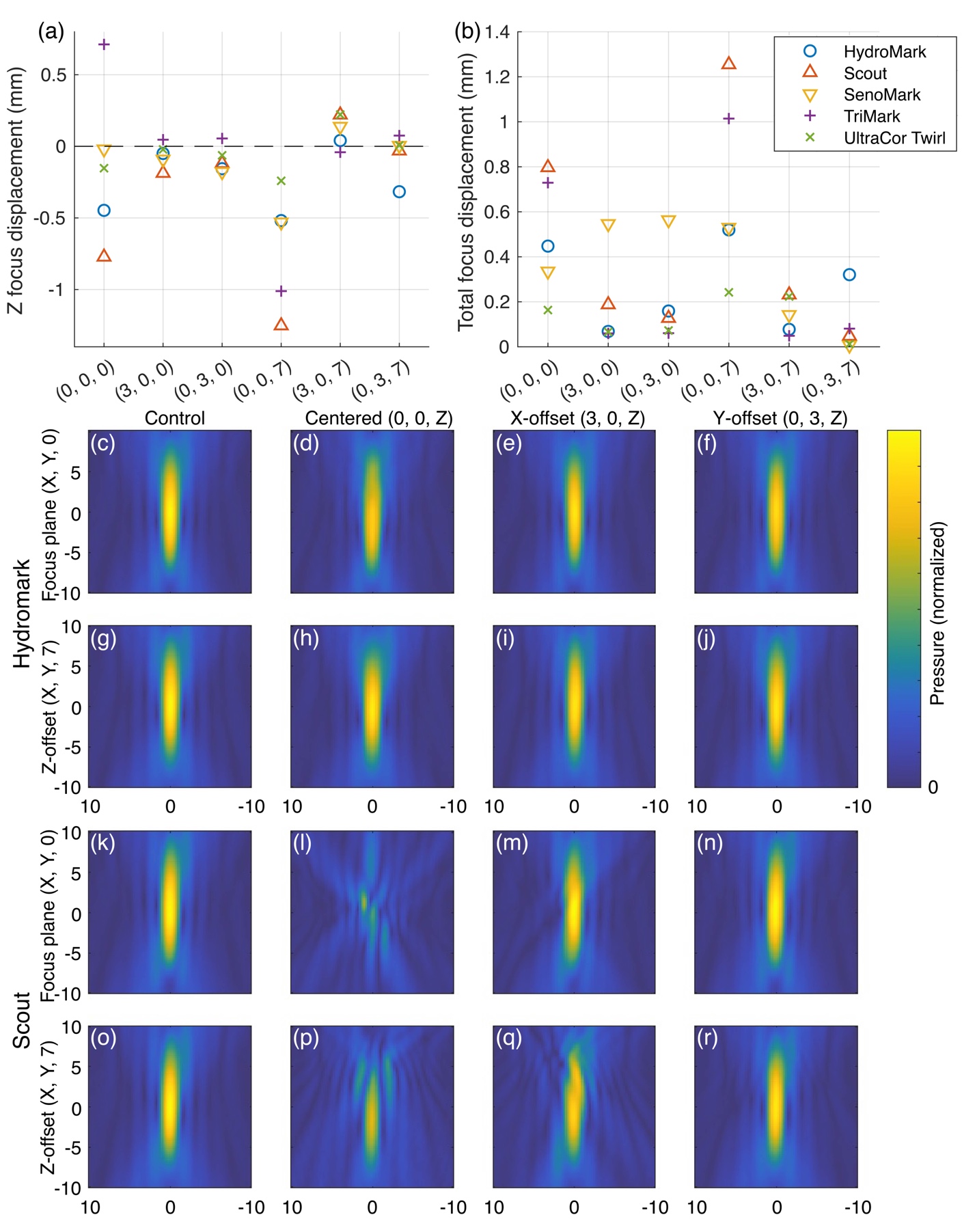
**

**Figure S8.** Hydrophone scan data sampled with and without a biopsy marker in the acoustic field. Biopsy markers are embedded in a thin (~2 mm) layer of gelatin. Plots show the displacement of the focus along the direction of propagation (a) and overall (b) relative to the paired control for each marker at six positions relative to the natural acoustic focus. In the normalized pressure maps (c-r), each row represents one of two sampling planes (see Figure 3). ‘Control’ column (c,g,k,o) acquired with no marker, only a thin layer of gelatin. Centered (d,h,l,p), X-offset (e,I,m,q), and Y-offset (f,j,n,r) columns reference the position of the marker as described in Figure 3. Pressure maps are normalized to the maximum pressure in the control volume for that row. (Compare Figure 8)

**
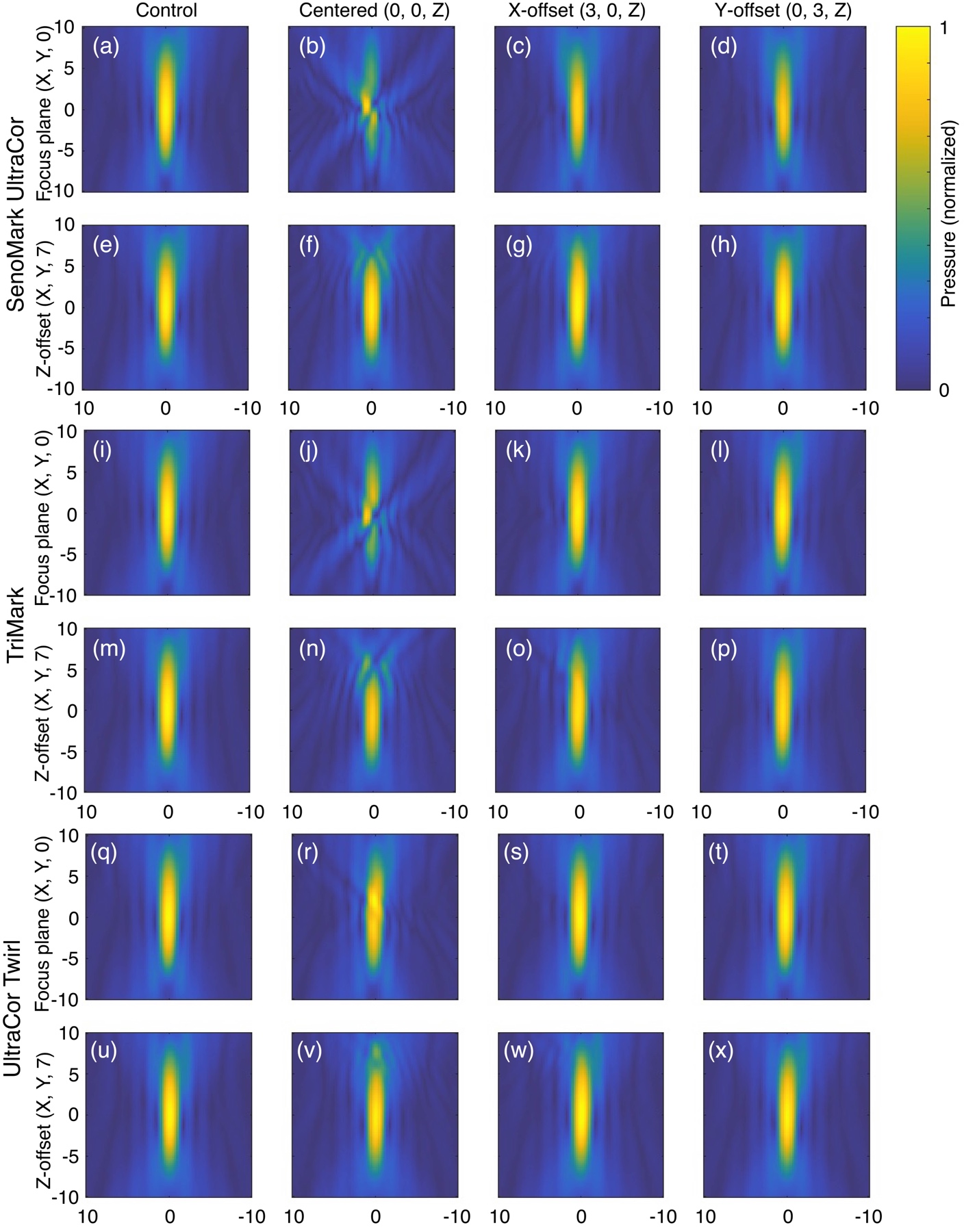
**

**Figure S9.** Hydrophone scan data sampled with and without a biopsy marker in the acoustic field. Biopsy markers are embedded in a thin (~2 mm) layer of gelatin. Each row represents one of two sampling planes (see Figure 3). ‘Control’ column acquired with no marker, only a thin layer of gelatin prepared from the same batch and at the same time as the respective samples. Centered, X-offset, and Y-offset columns reference the position of the marker relative to the axis of propagation during data acquisition, as shown in Figure 3. Pressure maps are normalized to the maximum pressure in the control volume for that row. (Compare Figure 8)
